# Deep-kidney: an effective deep learning framework for chronic kidney disease prediction

**DOI:** 10.1007/s13755-023-00261-8

**Published:** 2023-12-01

**Authors:** Dina Saif, Amany M. Sarhan, Nada M. Elshennawy

**Affiliations:** Department of Computers and Control Engineering, Faculty of Engineering, Tanta University, Tanta, Egypt

**Keywords:** Chronic kidney disease, CKD, CKD prediction, Health risk prediction, Deep learning, Deep ensemble

## Abstract

Chronic kidney disease (CKD) is one of today’s most serious illnesses. Because this disease usually does not manifest itself until the kidney is severely damaged, early detection saves many people’s lives. Therefore, the contribution of the current paper is proposing three predictive models to predict CKD possible occurrence within 6 or 12 months before disease existence namely; convolutional neural network (CNN), long short-term memory (LSTM) model, and deep ensemble model. The deep ensemble model fuses three base deep learning classifiers (CNN, LSTM, and LSTM-BLSTM) using majority voting technique. To evaluate the performance of the proposed models, several experiments were conducted on two different public datasets. Among the predictive models and the reached results, the deep ensemble model is superior to all the other models, with an accuracy of 0.993 and 0.992 for the 6-month data and 12-month data predictions, respectively.

## Introduction

Chronic kidney disease (CKD) refers to kidney damage caused by an inability to filter blood properly. The kidneys’ primary function is to filter extra water and waste from human blood and then excrete them through urine. That is, when a person has CKD, wastes accumulate in his body and cause symptoms such as back pain, diarrhea, nosebleeds, fever, rash, vomiting, and abdominal pain. As a result of the damage occurring gradually over time, it will affect the rest of the human body and lead to the emergence of many diseases. As the disease advances and reaches its final stages, it may result in death.

Because of the lack of early detection of these diseases, mortality from infection with many diseases has recently increased. As a consequence, many studies have emerged to address this issue, assist doctors, and reduce mortality through the use of advanced computer-based detection techniques. Early diabetes prediction, for example, has been proposed using the Random Forest and XGBoost algorithms [1]. Furthermore, for disease detection, a multi-layer perceptron and Random Forest algorithms were used [2, 3]. For predicting the likelihood of a human being a recent or future heart disease patient, a convolutional neural network model was developed [4]. For the prediction of coronary heart disease, an accuracy-based-weighted aging classifier ensemble algorithm was proposed [5].

CKD prediction using deep learning techniques, which is our main interest in this paper, is a very significant application of intellectual intelligent systems because it predicts the disease before it occurs, which greatly contributes to saving people’s lives. As an outcome, in order to defeat this dangerous disease, an effective mechanism for predicting CKD must be developed. Some studies focus on early detection, while only a few focus on predicting disease before it occurs. Multiple studies used Support Vector Machine and Artificial Neural Networks, Deep neural networks, an Ensemble algorithm, Extra tree, Random Forest, and Logistic Regression models to detect CKD at an early stage [6–11]. Furthermore, for CKD classification, the Density-based Feature Selection (DFS) with Ant Colony based Optimization (D-ACO) algorithm was proposed [12]. In terms of CKD prediction, Decision tree, Random forest, LightGBM, Logistic Regression, and CNN models have been developed to predict the disease 6–12 months in advance [13].

According to a massive amount of research in the machine learning field, two algorithms currently dominate this field: Ensemble and Deep Learning algorithms [14]. Deep learning is the gold standard of machine learning algorithms, and deep ensemble algorithms are a catch-all term for approaches that combine multiple deep learning classifiers to make a decision. As a result, in the current work, we use an ensemble algorithm in conjunction with ensemble and deep learning approaches. Deep learning techniques, on the other hand, are regarded as the most dominant and powerful player in a variety of machine learning challenges. It is primarily responsible for making a final prediction by locating hidden information in the massive dataset that doctors frequently obtain from patients. The deep learning model attempts to learn features that traditional techniques would not be able to extract. The use of this algorithm improves detection and prediction accuracy by avoiding the drawbacks of traditional learning techniques [15]. Deep learning techniques now outperform traditional classification techniques in terms of performance. Convolutional neural networks [16], long short-term memory [17], and many other techniques are used by deep learning algorithms to solve machine learning challenges. Over the last few years, many algorithms that combine ensemble algorithms and deep learning models have been developed in an attempt to improve predictive performance. The deep ensemble learning algorithm combines the benefits of both deep learning and ensemble learning to produce a final model with the best generalization performance [18].

In the case of kidney disease, scientists have attempted to detect it early or predict its occurrence. Disease detection implies that the patient already has the disease, whereas disease prediction implies that it will occur in the future. As a result, research in this area has been divided into two branches: detection and prediction. There are numerous studies in this field for the first type [13].

We faced the following challenges after reviewing the literature:

1. There isn’t enough data on CKD. The datasets for previous studies were based on medical tests. It does, however, consist of a small number of samples.
2. Previous research has focused on detecting the disease after it has occurred.
3. Due to a lack of data, the work in this field has not been fully explored.
4. There has only been one previous study that attempted to predict the disease in advance. However, the precision of this work was not high.
5. According to the previous challenges, the mortality rate of CDK disease is rapidly increasing.

As a result, in this paper, we will investigate several deep learning models as well as the ensemble approach to merge these models in order to fill a gap in the field. As a result, the following are the main contributions of this paper:

1. We propose two deep learning predictive models to predict CKD 6 or 12 months before disease occurrence, which are as follows:
  1.1 Convolutional Neural Networks (CNN) Model.
  1.2 Long Short-Term Memory (LSTM) Model.
2. We propose an ensemble model that uses the majority voting technique to combine three basic deep learning classifiers (CNN, LSTM, and LSTM-BLSTM) to improve classification performance.
3. For the task of CKD prediction, we train each model using two different public benchmark datasets. The first is to predict the disease 6 months ahead of time, while the second is to predict the disease 12 months ahead of time.
4. We assess the predictive models’ performance using various metrics to investigate the advantages and disadvantages of each. To demonstrate the strength of the proposed models, the results are compared to the results of the state-of-the-art work [13] using the same datasets.

In addition to the current section, section “Background and related work” presents previously developed approaches in risk detection and prediction for CKD, and classification ensemble techniques. The dataset is presented in section “Materials and methodologies” and the proposed models in described in detail. Section “Proposed models evaluation” evaluates the proposed predictive models, draws a comparative analysis, and discusses the prediction results. Section “Conclusion” concludes this paper.

## Background and related work

Many researchers proposed algorithms for health risk prediction for a variety of diseases in an effort to reduce mortality. Li et al. [19] forecasted the risk of hospital readmission for diabetic patients using a combination of collaborative filtering-enhanced and deep learning approaches in 2018. With 88.94% accuracy, the algorithm outperforms the Naïve Bayes, SVM, and decision tree algorithms. Later that year, Alam et al. [3] created a medical data classifier based on the Random Forest algorithm and feature ranking for ten different diseases. The proposed method was based on determining the significance of features for classification using various feature ranking techniques.

In 2020, Bikku et al. [2] proposed a multi-layer perceptron algorithm based on supervised learning methods to predict the risk of various diseases (breast cancer, diabetes, and heart disease) with a high degree of certainty. Following that, Shankar et al. [4] developed a new technique based on Naïve Bayes and KNN to predict the likelihood of a human being a recent or future heart disease patient. The prediction of coronary heart disease entered the competition as well, with the introduction of an accuracy-based-weighted aging classifier ensemble algorithm (AB-WAE) [5]. On two different datasets, this algorithm achieved 93% and 91% accuracy.

Since diabetes classification has occupied researchers’ minds for the riskiness of this disease, the Random Forest and XGBoost algorithms have been applied to the PIMA diabetes dataset for early prediction of this disease. XGBoost algorithm was superior to Random Forest by achieving 74.10% accuracy, while Random Forest achieved 71% accuracy [1]. Random Forest was proven to be superior to XGBOOST in CKD prediction reaching an accuracy of 100% as in [9, 11] using the CKD dataset [20].

### Risk detection and prediction for chronic kidney disease

Given the riskiness of kidney disease to human health, scientists have attempted to detect it early or predict its occurrence in advance. Disease detection implies that the patient already has the disease, whereas disease prediction implies that it will occur in the future. As a consequence, research has been divided into two types: detection and prediction. In aspects of the first type, most of them used the same dataset [20], beginning with CDK detection. Almansour et al. [6] used SVM and ANN to detect CKD at an early stage. The dataset was preprocessed, and then the missing values were replaced. Following that, ten fold cross-validation was used. This study concluded that ANN outperformed SVM in terms of accuracy, with accuracy up to 99.75%. The limitation of this study is that the number of samples is limited, which causes the problem of dimensionality. This problem was solved by employing the SVM algorithm. This study suggests that a deep learning technique be used to detect CKD.

Elhoseny et al. [12] developed an intelligent classification technique for CKD in the same year, called Density-based Feature Selection (DFS) with Ant Colony based Optimization (D-ACO). This technique solved the problem of increasing the number of features in medical data by removing redundant features, which greatly aided in the resolution of many issues such as low interoperability, high computation, and overfitting. Using this method, the author achieved 95% detection accuracy with only 14 of the 24 features.

During the same year, Kriplani et al. [7] proposed a deep neural network model to detect the absence or presence of CKD in its early stages. This model used cross-validation to avoid overfitting and outperformed other available techniques, reaching 97% accuracy when compared to Naïve Bayes, Logistic, Random forest, Adaboost, and SVM.

Following that, in 2020, Jongbo et al. [8] used an ensemble algorithm: Random Subspace and Bagging to achieve 100% accuracy on the previous dataset, which is appropriate for efficient CKD diagnosis. The data is preprocessed, then missing values are handled, and finally the data is normalized. This algorithm was based on majority voting between three base-learners: KNN, Naïve Bayes, and Decision Tree. This study demonstrated that combining the base classifiers improved classification performance. In the performance matrices, the proposed model outperformed individual classifiers, according to the experimental results. In most cases, the random subspace algorithm outperformed the bagging algorithm.

During the same year, Ekanayake et al. [9] proposed an efficient method for detecting CKD based on medical data, beginning with data prepossessing and then filling missing values with K Nearest Neighbors-imputer, which resulted in higher detection model accuracy. Finally, the classification method was used. They focused on the practical aspects of data collection, emphasizing the importance of combining domain knowledge in CKD detection using machine learning. Among the 11 classifiers tested, the authors demonstrated the superiority of extra tree and Random Forest classifiers in CKD detection: (logistic regression, KNN, SVC with a linear kernel, SVC with RBF kernel, Gaussian NB, decision tree classifier, XGB classifier, Adaboost classifier, and a classical neural network). The K-Nearest Neighbors-imputer is recommended for this study to handle missing values in other diseases. Furthermore, adding more features to the analysis is important, such as food types, water consumption, and genomics knowledge.

In addition, Gudeti et al. [10] distinguished the performance of several machine learning techniques in 2020 based on their accuracy in analyzing CKD and distinguishing between CKD and Non-CKD patients. To detect CKD, the authors used Logistic Regression, SVM, and KNN models. The SVM model outperformed the other techniques, achieving 99.2% accuracy. The main benefit of this research is that the detection process is quick, allowing doctors to start treating patients sooner and further categorizing the patient population in less time. They did, however, use a small dataset of 400 patients.

Later that year, in 2021, Chittora et al. [21] detected CKD using full or important features. Full features, correlation-based feature selection, Wrapper technique feature selection, least absolute shrinkage and selection operator regression LASSO, synthetic minority over-sampling technique with least absolute shrinkage and selection operator regression selected features, and synthetic minority over-sampling method using full features were used to calculate the results. C5.0, CHAID, ANN, linear support vector machine (LSVM), logistic regression (LR), random tree (RT), and KNN were also used as classifiers. Finally, with the full features in synthetic minority over-sampling technique, LSVM achieved the highest accuracy of 98.86%.

Following that, Senan et al. [11] used machine learning techniques to develop a diagnosis system to detect CKD to aid experts in early diagnosis. The mean and mode were used to replace the missing values. Recursive Feature Elimination was used to select the most important features (RFE). The dataset was divided into two parts: 75% for training and 25% for testing and validation. Following that, four machine learning algorithms were used: support vector machine (SVM), Random Forest (RF), k-nearest neighbors (KNN), and decision tree (DT). To achieve the best results, the parameters were tuned for all classifiers. Among these four classifiers, the Random Forest (RF) algorithm outperformed all other four techniques by achieving 100% accuracy.

Finally, Singh et al. [22] proposed a deep neural network in 2022. The missing values were replaced by the average of the associated feature, and the Recursive Feature Elimination (RFE) algorithm was used to select features. The key parameters in the study were Specific Gravity, Hemoglobin, Red Blood Cell Count, Creatinine Levels, Packed Cell Volume, Albumin, and Hypertension (RFE). Following that, the selected features were fed into five classifiers (Deep neural network DNN, Naïve Bayes classifier, KNN, Random Forest, and Logistic regression). DNN outperformed all other models in terms of accuracy. The size of the dataset is a limitation of both the proposed algorithm and previous studies. The next step in this research will be to collect more sophisticated and representative CKD data to detect disease severity. The authors intend to use the proposed model on medical data containing the night urination, acid–base parameters, inorganic phosphorus concentration, and hyperparathyroidism features.

Concerning the second type of investigation, disease risk prediction, the first pioneering technique was proposed in 2021, which concerned CKD prediction as opposed to previous searches, which concerned CKD detection [13]. The primary goal of this study was to forecast the occurrence of CKD 6–12 months before disease onset using Taiwan’s National Health Insurance dataset [23]. The predictive model was developed using comorbidity, demographic, and medication data from patients over a 2-year period. For 12-month and 6-month predictions, the CNN model had the best AUROC of 0.954 and 0.957, with accuracy of 88% and 89%, respectively. While, the most important predictors were: gout, diabetes mellitus, age and medications such as angiotensin and sulfonamides. Table 1 summarizes the recent health risk prediction research for CKD.

**Table 1:** Summary of recent health risk detection and prediction models for CKD

Paper	Detection/prediction	Dataset	Algorithm	Weaknesses	Strengths	Highest accuracy
Almansour-2019 [6]	Detection	CKD dataset-1 [20]	SVM-ANN	The number of samples is limited, objective is detection	Comprehensive comparison between SVM and ANN, promising results	ANN 99.75%
Elhoseny-2019 [12]	Detection	CKD dataset-1 [20]	Density-based Feature Selection (DFS) with Ant Colony based Optimization (D-ACO)	Complex system, no comparison to state-of-the-art, objective is detection	Using few features	95%
Kriplani-2019 [7]	Detection	CKD dataset-1 [20]	Deep neural Network, SVM, Naïve Bayes, Adaboost and Logistic regression	Small dataset, no comparison to state-of-the-art, objective is detection	Novel approach, high accuracy, implementation of the model is available which makes it reproducible	Deep Neural Network 97%
Jongbo-2020 [8]	Detection	CKD dataset-1 [20]	Ensemble: KNN, NB, DT	Complex system, no comparison to state-of-the-art, objective is detection	Ensemble approach, outperforms existing methods	100%
Ekanayake-2020 [9]	Detection	CKD dataset-1 [20]	Using 11 different classifiers	Small dataset, objective is detection	Evaluates 11 machine learning algorithms, high accuracy	Extra tree classifier and Random Forest 100%
Gudeti-2020 [10]	Detection	CKD dataset-1 [20]	SVM, LR and KNN	Small dataset, no discussion of the impact of different feature selection methods on the performance, objective is detection	Few features, the proposed approach is reproducible	SVM 99.2%
Chittora-2021 [21]	Detection	CKD dataset-1 [20]	C5.0, CHAID, ANN, LSVM, LR, RT and KNN	Small dataset, objective is detection	Evaluates seven algorithms, identifies important features	LSVM 98.86%
Senan-2021 [11]	Detection	CKD dataset-1 [20]	SVM, RF, KNN, DT	Small dataset—no comparison to state-of-the-art methods, detection only	High accuracy, using (RFE) to select the most important features, tuning the parameters of the classifiers	Random Forest 100%
Singh-2022 [22]	Detection	CKD dataset-1 [20]	DNN, NB, KNN, RF, LR	Small dataset, objective is detection	High accuracy, using (RFE) to select the most important features, The key parameters are specified	DNN 100%
Krishnamurthy-2021 [13]	Prediction	CKD dataset-2 [23]	CNN, BLSTM, LightGBM, LR, RF, DT	Low accuracy	Large and diverse dataset based on comorbidity and demographical data, objective is predicting the disease in advance	CNN
						89% (6 months)
						88% (12 months)

### Classification ensemble techniques

Ensemble techniques are considered state-of-the-art methodologies for solving problems in a wide range of machine learning applications. The intuitive motivation for ensemble stems from human nature and the proclivity to gather disparate viewpoints and integrate them in order to make a complex decision. This idea depends on integrating multiple base-learners to obtain a classifier that outperforms them all using one of the combination algorithms: [Average Ensemble (AE), Weighted Average Ensemble (WAE), and Majority Voting Ensemble (MVE)]. In recent years, machine learning researchers have demonstrated through hands-on experimental research that combining the outputs of multiple classifiers improves the performance of a single classifier [18]. The ensemble technique has been used in a variety of applications, including disease detection and prediction, due to its impact on several machine learning challenges [5, 8, 24, 25]. The ensemble technique’s main idea is to maximize predictive performance by combining the strengths of multiple individual classifiers. In other words, the goal of deep ensemble models is to create a model that incorporates the advantages of both ensemble and deep models.

Recently, there have been some issues with using an individual classifier, such as overfitting, class imbalance, concept drift, and the curse of dimensionality, which cause a single classifier prediction to fail [26]. As a result, this new method has emerged in scientific research to address these issues. The predictive accuracy improves by using this algorithm in different machine learning challenges. The main idea of any ensemble learning is to use a combination function F to combine a set k of individual classifiers, *c*_1_, *c*_2_, …, *c*_*k*_, to predict a single output. Given a dataset of size n and features of dimension m, *D* = {(*x*,*y*)}, 1 ≤ i ≤ n, x_i_ ∈ R^m^, the output’s prediction of this method is shown in Eq. (1) [27].

1 $${y}_{i}=\varnothing \left({x}_{i}\right)=f\left(c1.c2.. ck\right)$$

In this section, we will examine the most common Ensemble techniques that are commonly used in many machine learning applications, as well as some literature reviews on using ensemble techniques in health risk prediction.

#### Average ensemble (AE)

This technique demonstrated its high efficiency in scientific research. The main idea behind the techniques is that the final prediction is calculated by taking the average of the individual learners’ outputs. This average is calculated directly from the outcomes of individual learners or by applying the softmax function to the forecasting probabilities of the classes, as shown in Eq. (2). The performance of this technique is improved because of variance among the models is reduced [18].

2 $${P}_{i}^{j}=softmax({O}_{i})=\frac{{O}_{j}^{i}}{\sum_{K=1}^{K}\mathrm{exp}({O}_{j}^{i})}$$

where P_i_^J^ is the probability of the outcome of the ith unit on the jth base learner, ${O}_{j}^{i}$ is the output of the ith unit of the jth base learner and K is the number of the classes. This approach is appropriate when individual performance is proportional [28]. On the other hand, it is not appropriate when individual classifier performance is grossly disproportionate. The overall performance will be reduced in this case due to the influence of weak learners.

Because this technique does not take into account the performance of individual models, all models have the same weight. The previous method has the limitation that the results of the weak base classifier will have an adverse effect on the final model output. To avoid this problem, the Weighted Average Ensemble (WAE) is proposed, which provides sorted weights for models based on their efficiency.

#### Weighted average ensemble (WAE)

The previous approach is appropriate when the performance of the individuals are proportional [28]. On the other hand, it isn’t appropriate when the individual learners’ performances are absolutely disproportionate. In this case, the overall performance will be reduced according to the influence of weak learners. To avoid this problem, the (WAE) is proposed, which provides sorted weights for models based on their efficiency. It is thought to be an extension of the previous method, in which the final prediction value is obtained by calculating the average of all the base classifiers’ predictions. In contrast, in the weighted average, each data point is assigned a pre-defined weight to indicate its importance in the prediction, and the final prediction value is calculated by taking the weighted average of all the data points into account. Each classifier in the ensemble contributes to the final prediction based on its weight in this technique. The final prediction for class label prediction is calculated using the mode of the individuals’ predictions. For the class probability prediction, the final prediction is calculated using argmax of the summed probabilities of each class label [29].

#### Majority voting ensemble (MVE)

In the research field, this technique is regarded as the most widely used approach in the ensemble technique. This technique, similar to the previous ones, combines the outputs of individual learners. Instead of calculating the average of the probability results, (MVE) counts the votes of individual classifiers and predicts the final class based on the majority of votes [18]. The main advantage of this technique is that it is less biased towards the outcome of a specific individual learner because the majority vote count relieves the influence; additionally, the influence of weak learners is no longer significant. The majority voting rule comes in three varieties:

(i) Unanimous voting, in which all individual classifiers agree on the prediction;
(ii) Simple majority voting, in which the prediction must be supported by at least 51% of all classifiers; and
(iii) Majority or plurality voting, in which individual learners’ votes are counted and the final prediction is calculated based on the majority of votes. The majority voting rule improves prediction performance.

This model caught the interest of scientists and researchers, and it is now used in a variety of applications in health risk detection and prediction for a variety of diseases.

#### Literature review of using ensemble in disease detection

This section examines the literature on ensemble learning in disease detection, with machine learning or deep learning as individual classifiers. Using ensemble learning, Raza et al. [30] created a model for detecting heart disease that is both reliable and accurate. The majority voting rule was used to combine the results of three classification algorithms [logistic regression (LR), multilayer perceptron (MLP), and Naïve Bayes (NB)] in this paper. The proposed ensemble method achieved classification accuracy of 88.88%, which is superior to any other base classifier.

Following that, Atallah et al. [24] presented an ensemble method based on the majority voting technique in the same field. This method combined Stochastic Gradient Descent (SGD), KNN, Random Forest, and Logistic Regression to provide doctors with greater dependability and accuracy. Finally, using the hard voting ensemble model, this technique achieved 90% accuracy. Yadav et al. [31] used various ensemble techniques on 10 biomedical datasets [32]. These techniques performed competitively against individual classifiers. The highest AUC was achieved using the average ensemble and the Rank Average Ensemble (RAE) in most datasets.

Individual classifiers were outperformed by these techniques. In most datasets, the average ensemble and the Rank Average Ensemble (RAE) produced the highest AUC. Similarly, Tao Zhou et al. [33] proposed an ensemble deep learning model called (EDL COVID) to detect COVID 19 in lung CT images, employing a relative majority vote algorithm with 99.05% accuracy. Before employing the Ensemble technique, the base models were built using ResNet, GoogleNet, and AlexNet. In terms of performance and detection speed, the EDL COVID classifier outperformed the single classifiers. Similarly, Chandra et al. [34] used the majority voting ensemble technique to create a two-phase classification system [normal vs. abnormal (phase-I) and COVID-19 vs. pneumonia (phase-II)]. The obtained precision for Phase-I and Phase-II was 98.062% and 91.329%, respectively.

Neloy et al. [25] proposed an ensemble model to achieve an excellent result in heart disease prediction. Among the baseline models used in their proposed work are (Random Forest, Decision Tree, and Naïve Bayes). The combining process, which used the Weighted Average Ensemble technique, achieved 100% accuracy on training and 93% accuracy on testing [25].

Using voice recordings of 50 patients and 50 healthy people, Hire et al. proposed an ensemble algorithm of CNNs for detecting Parkinson’s disease. The publicly available database obtained from PC-GITA was used. The base classifier was trained using a multiple-fine-tuning method. Each vowel was trained and tested separately, then a tenfold validation was performed to test the models. The proposed approach was soundly able to differentiate between the voices of patients and the healthy people for all vowels. The proposed model achieved 99% accuracy, 93.3% specificity, 86.2% sensitivity, and 89.6% AUC. The monitoring of the patients can be applied online without needing additional hardware.

Table 2 summarizes the ensemble disease detection techniques, the dataset used in the experiments, and the highest accuracy.

**Table 2:** Previous ensemble models for various diseases detection

Paper	Dataset	Algorithm	Weaknesses	Strengths	Highest accuracy
Raza-2019 [30]	Heart disease dataset–statlog [35]	MVE	No evaluation on different datasets	Achieving promising results	88.88%
Atallah-2019 [24]	Heart disease dataset [32]	MVE	No evaluation on different datasets	Simple and effective majority voting ensemble method, promising results on real-world dataset, reproducible model, applicability	90%
Yadav-2019 [31]	Breast Cancer Wisconsin (Original) (683 samples)	AE–MVE-WAE	Each of the datasets contains a small number of samples	Evaluates a variety of classifiers on benchmark dataset, discusses advantages and disadvantages of ensemble and single classifiers	(AE) 0.9998 AUC
	Breast Cancer Wisconsin (Diagnostic) (569 samples)				(AE) & (RAE) 100% AUC
	Haberman’s Survival Dataset (306 samples)				(AE) 0.636
	Heart Disease Dataset (Hungarian) (261 samples)				(AE) 0.8994
	Indian Liver Patient Database (579 samples)				(AE) 0.7892
	Mammographic Mass Dataset (830 samples)				(AE) 0.8708
	Single-Proton Emission Computed Tomography (SPECT) (267 samples)				(WAE) 0.8166
	SPECTF Heart-imaging Dataset (267 samples)				(RAE) 0.8166
	Statlog (Heart) Dataset (270 samples)				(RAE) 0.9272
	Vertebral Column Dataset (310 samples				(AE) & (RAE) 0.9504
Tao Zhou-2021 [33]	The data is available from the author upon request	MVE	No evaluation with different datasets	Achieving high accuracy on benchmark dataset, Comparing the results with deep learning models	99.05%
Chandra-2021 [34]	COVID‐Chestxray [36]	MVE	No comparison with other state-of-the-art COVID-19 detection methods using chest X-ray images	High accuracy, displaying statistical analysis	98.062% Phase-I 91.329% Phase-II
Neloy-2022 [25]	Heart disease dataset [37]	WAE	Lack of evaluation on different types of datasets	Novel weighted average ensemble technique achieves promising results on real-world dataset	100% training 93% testing
Hireš-2022 [38]	Parkinson’s disease	MV	Lack of evaluation on different types of datasets	Novel CNN ensemble technique achieves promising results on real-world Parkinson’s disease dataset	99%

## Materials and methodologies

### Dataset description

The datasets publicly released from Taiwan’s National Health Insurance Research Database (NHIRD) [39] is used in our study. The author of [13] compiled patient data into CSV files in order to predict CKD disease 6–12 months in advance [23]. Each patient’s data was saved for 2 years, and it consisted of 965 comorbidities denoted by ICD 9 codes (International Classification of Diseases) and 537 medications denoted by Anatomical Therapeutic Chemical code (ATC codes) for 6 months’ data. It includes 967 comorbidities and 539 medications for a 12-month period of data, as well as the patient’s age and gender. There is also a class label that represents CKD. Each patient is labelled with a binary number (0 means the patient will not get CKD after the specified period, while 1 means he will get CKD after a certain period). A total of 90,000 patients are analyzed, divided into 18,000 with CKD and 72,000 without CKD diagnose separately for each dataset.

**Fig. 1:**
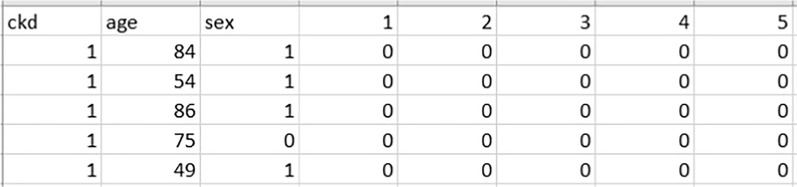
Part of a sample of the used dataset

Figure 1 represents a part of a sample of the used dataset [39]. As we see in the figure, “ckd” represents the target column, while “age” represents the patient’s age. The “sex” column denotes the patient’s gender, while (1–5) represents the “ICD_9” code, which represents a number of diseases, including Cholera disease, Typhoid and paratyphoid fevers, Salmonella infections, Shigellosis, and poisoning. For each patient, a zero indicates that he was not infected with the disease throughout the observation period of 2 years; otherwise, it indicates that he was infected.

### Methodologies

The prediction problem is treated as a classification problem, with the model’s output being either 0 or 1. (0 means the patient will not get CKD after the specified period, while 1 means he will get CKD after a certain period). We present the architecture of the three proposed predictive models for chronic kidney disease prediction in this section (CKD). Because there has only been one work directed toward solving this problem [13], we intend to explore different models for the problem using deep learning models in our presented work. Deep learning algorithms are the gold standard in machine learning. It is useful in a variety of applications when analyzing large amounts of data. 90,000 patients were analyzed in our two datasets, with 1504 features for 6 months’ data and 1508 features for 12 months’ data. As a result, this algorithm can predict CDKs by uncovering hidden information in the large dataset that doctors frequently obtain from patients. The deep learning model attempts to learn features that traditional techniques cannot extract. Using the same datasets, traditional machine learning techniques did not achieve the desired accuracy. As a result, the use of this algorithm improves detection and prediction accuracy by avoiding the drawbacks of traditional learning techniques. The first two predictive models are deep learning models: CNN and LSTM, while the third is an ensemble model composed of three different deep learning models.

Convolutional neural networks (CNNs) and long short-term memory networks (LSTMs) were chosen in the context of CKD prediction due to their unique advantages. When working with tabular data that may contain spatially important information in the context of CKD, it is essential to be able to capture spatial patterns, which CNNs are good at doing. However, LSTMs are excellent at modelling temporal relationships, which makes them a good choice for examining sequential data from the CSV file that can capture the development of CKD-related traits over time. A comprehensive approach to feature extraction that takes into account the dataset’s spatial and temporal dimensions is made possible by combining CNNs with LSTMs. We use these models to enhance prediction accuracy by capturing comprehensive patterns and relationships within the data.

**Fig. 2:**
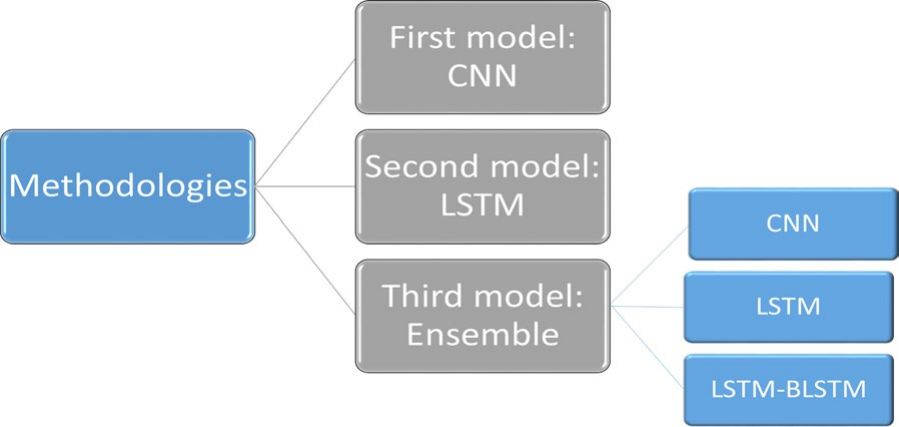
Schematic diagram for the methodologies

The methodologies adopted in this work are depicted schematically in Fig. 2. Each model is trained twice for different prediction tasks (the first time using 6 months of data and the second time using 12 months of data). Each model’s input is a CSV file containing 90,000 samples with 1504 features for 6 months’ data and 1508 features for 12 months’ data. The input features have been reshaped before applying the model to match the model requirements, while the output is a binary number that represents the class. The same model structures are used for both benchmark datasets, but the input layer is reshaped differently due to the difference in the number of features in each.

#### Convolutional neural network (CNN)-CDK predictive model

**Fig. 3:**
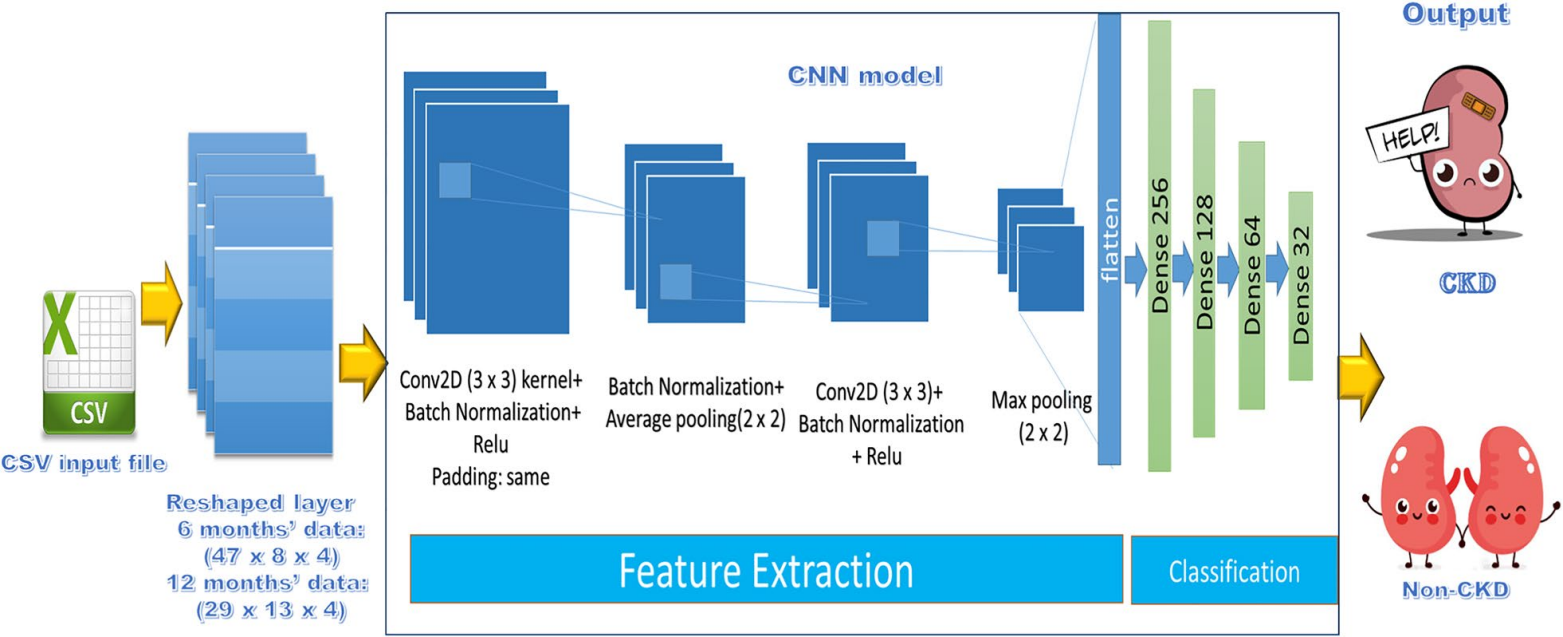
Proposed CNN predictive model for CKD prediction

CNN-based models have demonstrated robust performance in a variety of research applications. As a result, the first proposed predictive model in our work, as illustrated in Fig. 3 and Table 3, is based on this robust model. The input layer receives 1504 features for 6 months’ data, which are then reshaped to (47 × 8 × 4). While the input layer receives 1508 features for a 12-month period data, which is then reshaped to (29 × 13 × 4) to match the network configuration. The output layer is made up of a neuron that determines the class (1 representing CKD and 0 representing Non-CKD).

**Table 3:** Architecture details of proposed CNN predictive model for CKD prediction

Layer (type)	Output shape	Param #
batch_normalization	(None, 47, 8, 4)	16
conv2d (Conv2D)	(None, 47, 8, 512)	18,944
batch_normalization_1	(None, 47, 8, 512)	2048
average_pooling2d (AveragePooling2D)	(None, 23, 4, 512)	0
Conv2d_1 (Conv2D)	(None, 21, 2, 256)	1,179,904
batch_normalization_2	(None, 21, 2, 256)	1024
max_pooling2d (MaxPooling2D)	(None, 10,1, 256)	0
dropout (Dropout)	(None, 10,1, 256)	0
flatten (Flatten)	(None, 2560)	0
dense (Dense)	(None, 256)	655,616
dense_1 (Dense)	(None, 128)	32,896
dropout_1 (Dropout)	(None, 128)	0
dense_2 (Dense)	(None, 64)	8256
dropout_2 (Dropout)	(None, 64)	0
dense_3 (Dense)	(None, 32)	2080
dense_4 (Dense)	(None, 1)	33
Total params: 1900, 817 Trainable params: 1899, 273 Non-trainable params: 1544		

The activation function is applied to obtain the output as shown in Eq. (3)

3 $${h}^{k}=f\left({W}^{k}*x+{b}^{k}\right)$$

where *h*^*k*^ represents the output feature map, $x$ is the input, *b*^*k*^ and *W*^*k*^ are the bias and the weight of the k neuron, respectively [40].

The average pooling technique has been adopted, with a kernel size of 2. This technique works by moving a pool with a specific size over the incoming input, then calculating the average value in each one. Moreover, the max pooling technique has been utilized, with a kernel size of 2. Furthermore, the dropout mechanism is used to avoid overfitting and improve network performance.

#### Long short-term memory (LSTM)-CDK predictive model

LSTM is a type of deep learning network model that is frequently used in time-series signals analysis. The most significant advantages of this model are [41]: it has a higher accuracy in long-term dependency problems than Recurrent Neural Network (RNN). Furthermore, vanishing gradients problems can be solved using memory blocks using this technique.

The LSTM unit consists of an input gate I_t_, an output gate O_t_ and a forget gate F_t_. The three gates’ activations are computed using the subsequent equations [42]:

4 $${I}_{t}=\sigma ({W}_{i}{X}_{t}+ {R}_{i}{H}_{t-1} + {b}_{i})$$

5 $${F}_{t}=\sigma ({W}_{f}{X}_{t}+ {R}_{f}{H}_{t-1} + {b}_{f})$$

6 $${O}_{t}=\sigma ({W}_{o}{X}_{t}+ {R}_{o}{H}_{t-1} + {b}_{o})$$

The sigmoid activation function and the current input are represented as σ and X_t_ respectively. The input weights are denoted as W_i_, W_f_ and W_o_ while b_i_, b_f_ and b_o_ are the bias. Whilst the recurrent weights are symbolized as R_i_, R_f_ and R_o_. The output of the previous block is represented as H_t−1_. The modified new memory $\overline{C }$_t_ is computed as in Eq. (7):

7 $${\overline{C} }_{t}=tanh({W}_{t}{X}_{t}+ {R}_{t}{H}_{t-1} + {b}_{t})$$

where tanh(·) represents the hyperbolic tangent function. Whilst R_t_ and W_t_ denote the recurrent weight and input weight respectively. The computation of the current memory cell C_t_ is illustrated as in Eq. (8):

8 $${C}_{t}={F}_{t} \odot {C}_{t-1} + {I}_{t}\odot {\overline{C} }_{t}$$

where C_t−1_ represents the previous memory cell while ⊙ indicates the element-wise multiplication operation. To calculate the LSTM output H_t_, the following equation is used:

9 $${H}_{t}={O}_{t} \odot { tanh(C}_{t })$$

**Fig. 4:**
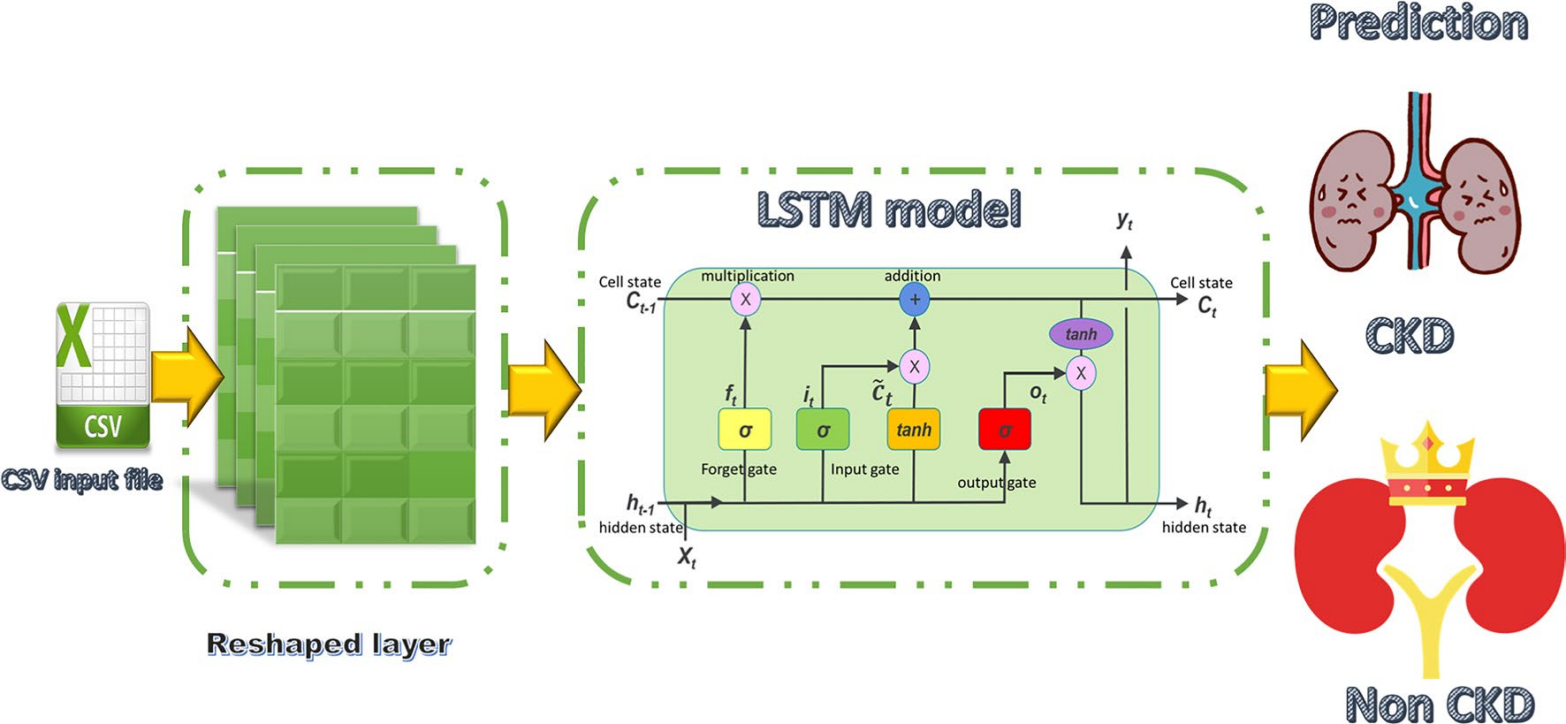
Proposed LSTM predictive model for CKD prediction

The input layer receives 1504 features for 6 months’ data, then reshaped to (47 × 32). While it is reshaping to (52 × 29) for 12 months’ data to match the network configuration. The structure diagram and the architecture of LSTM are illustrated in Fig. 4 and Table 4.

**Table 4:** Architecture proposed LSTM predictive model for CKD

Layer (type)	Output shape	Param #
lstm (LSTM)	(None, 47, 500)	1,066,000
lstm_1 (LSTM)	(None, 200)	560,800
flatten (Flatten)	(None, 200)	0
dense (Dense)	(None, 128)	25,728
dropout (Dropout)	(None, 128)	0
dense_1 (Dense)	(None, 64)	8256
dropout_1 (Dropout)	(None, 64)	0
dense_2 (Dense)	(None, 32)	2080
dense_3 (Dense)	(None, 1)	33
Total params: 1, 662, 897 Trainable params: 1, 662, 897 Non-trainable params: 0		

#### Deep ensemble predictive model (DEM)

**Fig. 5:**
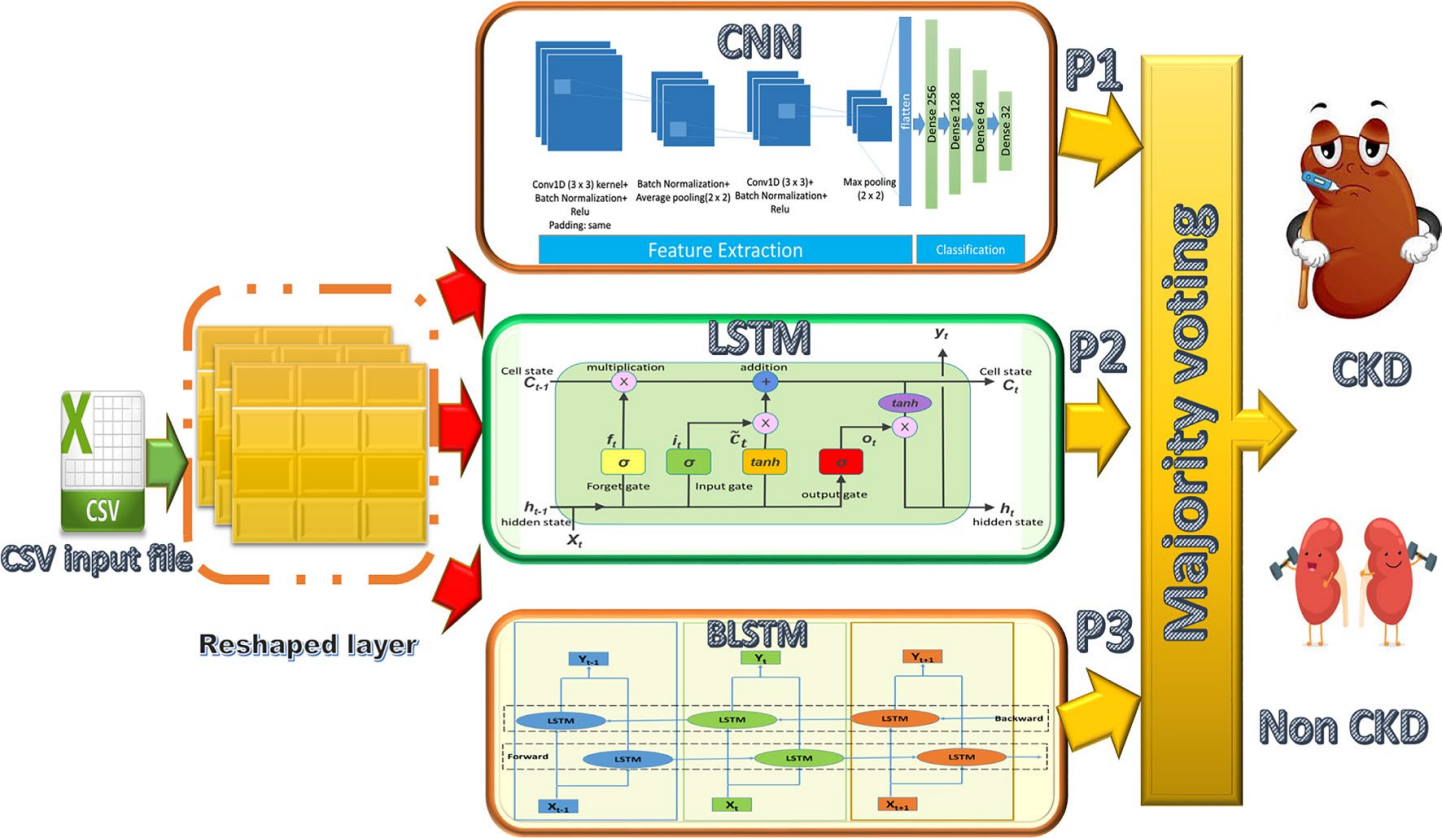
Structure of the proposed ensemble CDK predictive model

Ensemble learning methods are usually used to improve prediction performance when a single classifier is insufficient to achieve a high level of performance. The main idea behind this predictive model is to aggregate a group of different individual classifiers to improve performance by combining a weak classifier with a strong classifier to increase the efficiency of the weak learner. In our proposed ensemble model, we combine CNN, LSTM, and LSTM-BLSTM models to produce an effective computational model for CKD prediction based on majority voting ensemble, as shown in Fig. 5. The majority voting ensemble was chosen due to its robustness and because it is less biased towards the outcome of a particular individual learner. Furthermore, the influence of weak learners is no longer significant, and finally, its impressive results in disease detection are documented in the literature [18, 24, 25, 30, 31, 33, 34].

The following subsections provide details on each individual model in the ensemble model.

##### First model in the ensemble: CNN model_2

The structure of the ensemble’s first model is a CNN model, referred to as CNN model_2, as depicted in Fig. 5 and Table 5. The figure illustrates the application of a 1D CNN in our proposed model to generate a fast, generic, and highly accurate CKD predictive model. The 1D convolution is represented by the following equation:

10 $$x_{k}^{l} = b_{k}^{l} + \sum\nolimits_{i = 1}^{Ni - 1} {conv1D\left( {w_{ik}^{l - 1} ,s_{i}^{l - 1} } \right)}$$

where ${b}_{k}^{l}$ is the bias for layer l of the kth neuron, ${x}_{k}^{l}$ is the input for the same layer, ${s}_{i}^{l-1}$ is the output of the ith neuron at layer l − 1, ${w}_{ik}^{l-1}$ is the kernel (filter) from layer l − 1 to layer l.

**Table 5:** Architecture of LSTM-BLSTM model

Layer	Output shape	Param #
lstm (LSTM)	(None, 47, 500)	1,066,000
bidirectional (Bidirectional)	(None, 200)	480,800
flatten (Flatten)	(None, 200)	0
dense (Dense)	(None, 128)	25,728
dropout (Dropout)	(None, 128)	0
dense_1 (Dense)	(None, 64)	8256
dropout_1 (Dropout)	(None, 64)	0
dense_2 (Dense)	(None, 32)	2080
dense_3 (Dense)	(None, 2)	66
Total params: 1, 582,930 Trainable params:1, 582,930 Non-trainable params: 0		

The output, ${y}_{k}^{l}$, can be calculated by passing the input ${x}_{k}^{l}$ through the activation function as follows:

11 $$y_{k}^{l} = f\left( {x_{k}^{l} } \right)$$

The back-propagation algorithm (BP) is then used to reduce the output error. This algorithm works its way backwards from the output layer to the input layer. Consider the output layer (L). The number of classes is represented by NL, and for an input vector p, the target and output vectors are represented by ${t}_{i}^{p}$ and [${y}_{1}^{L}$,⋯,${y}_{NL}^{L}$], respectively. As a result, the mean-squared error (MSE), *E*_*p*_, can be computed as follows:

12 $$E_{p = MSE} \left( {{}_{t}^{p} ,\left[ {y_{1}^{L} , \ldots y_{NL}^{L} } \right]} \right) = \sum\nolimits_{i = 1}^{NL} {\left( {y_{i}^{L} - t_{i}^{p} } \right)} ^{2}$$

The derivation is used, and the various gradients of the neurons are computed recursively. As a result, the network’s weights are updated until the least error is reached.

This model is composed of Conv1D, avg_pooling, Conv1D, max_pooling, dropout, flatten, dense 256, dense 128, dropout, dense 64, dropout, dense 32 which is finally connected to another dense layer for CKD prediction.

##### Second model in the ensemble: LSTM model_2

We use LSTM in our model to avoid the vanishing gradient problem and to build a high-performance computational framework predictive model. The same equations as mentioned in section “Deep ensemble predictive model (DEM)” are used. The model is made up of an LSTM layer with 500 hidden units. Then, another LSTM layer with 200 hidden units is added. The previous layers are followed by a dense layer of 128 neurons. A dropout is used, followed by a second dense layer of 64 neurons. The dropout is used again to avoid overfitting and improve model performance. Following these layers is a dense layer of thirty-two neurons, which is finally connected to another dense layer for CKD prediction.

##### Third model in the ensemble: LSTM-BLSTM model

As shown in Fig. 5 and Table 5, the third model in the ensemble is a hybrid model that combines LSTM and BLSTM in an attempt to improve the performance of the ensemble models. A Bidirectional LSTM (BLSTM) is an enhanced version of LSTM. It is made up of two LSTMs that work in opposite directions (forward and backward). The forward direction is represented by ${h}_{t}^{f}$ that denotes the input in ascending order, i.e., t = 1, 2, 3, …, T. The opposite direction is represented by a backward hidden layer called ${h}_{t}^{b}$, which represents the input in descending order, i.e., t = T, …, 3, 2, 1. Finally, ${y}_{t}$ is generated by combining ${h}_{t}^{f}$ and ${h}_{t}^{b}$. The BLSTM model is represented by the following equations:

13 $$h_{t}^{f} = H\left( {W_{xh}^{f} X_{t} + W_{hh}^{f} h_{t - 1}^{f} + b_{h}^{f} } \right)$$

14 $$h_{t}^{b} = H\left( {W_{xh}^{b} X_{t} + W_{hh}^{b} h_{t + 1}^{b} + b_{h}^{b} } \right)$$

15 $$y_{t} = W_{hy}^{f} h_{t}^{f} + W_{hy}^{b} h_{t}^{b} + b_{y}$$

where W is a weight matrix (${W}_{xh}^{f}$ is a weight that connects input (x) to the hidden layer (h) in the forward direction, while ${W}_{xh}^{b}$ is the same but in the backward direction). ${b}_{h}^{f}$ is a forward direction bias vector, whereas, ${b}_{h}^{b}$ is a backward direction bias vector, the out is symbolized by ${y}_{t}$ [43].

## Proposed models evaluation

The experiments are carried out using a publicly available dataset [23] that contains two different types of samples. The first one represents CKD prediction over 6 months earlier, while the other represents CKD prediction over 12 months earlier. Each one involves 90,000 samples, with 80% of them used for training and 20% for validation. The aforementioned models were implemented using Python 3 involving the Keras framework running on Google Colab using a GPU on processor: (Intel(R) Xeon(R) CPU @ 2.20 GHz) with 13 GB RAM.

The classification process used by the trained deep learning models is applied on the test dataset. As for the first two models (CNN and LSTM) models, the new sample is fed to each model to generate the final prediction. On the other hand, when a test sample is fed to the proposed Ensemble model, it is first distributed to all individual models. Next, each classifier produces a prediction. After that, the majority voting technique is applied to all base classifiers’ results to generate the final prediction.

### Performance metrics

To compare the models’ performance, four commonly used performance evaluation metrics were used: true negative (TN), true positive (TP), false negative (FN), and false-positive (FP). Furthermore, five metrics are used in the evaluation: Recall, Precision, Accuracy, F1_score, and specificity which are calculated as given in Eqs. (16)–(20). A recall is the number of positive instances predicted from the total number of positive instances; it is also known as sensitivity or true positive rate. Precision, also known as Positive Predictive Value, is the number of instances predicted as positive out of the total number of samples predicted as positive. Accuracy is defined as the number of correctly predicted instances divided by the total number of instances. F1-score combines Precision and Recall into a single metric using their harmonic mean. The number of instances predicted as negative out of the total number of negative instances is referred to as specificity.

16 $$Recall \left( {Sensitivity} \right) = \frac{TP}{{TP + FN}}$$

17 $$Precision =\frac{TP}{TP+FP}$$

18 $$Accuracy=\frac{TP+TN}{TP+TN+FP+FN}$$

19 $${F}_{1}\_score=2* \frac{Precision \times Recall}{Precision + Recall}$$

20 $$Specificity =\frac{TN}{TN+FP}$$

where TP denotes true positive or correctly classified positive class, TN denotes true negative or correctly classified negative class, FP denotes false positive or incorrectly classified positive class, and FN denotes false negative or incorrectly classified negative class.

To assess the impact of the proposed deep ensemble approach on prediction results, we ran several experiments on the aforementioned benchmark datasets and compared the ensemble’s performance to all individual models. Finally, we present all experimental results and compare them to previous recent results [13].

### Experimental results and comparative analysis

This section presents the results of the proposed models, as well as the performance evaluation metrics mentioned in section “Performance metrics”. Tables 3 and 4 show the structure of the CNN and LSTM models used in this study. The activation function of the convolution layer and dense layer is (ReLU: Rectified Linear Unit), and the model employs the (Adamax) optimizer with a learning rate (lr) of 0.0009, with all other parameters set to default values.

Figure 5 depicts the structure of the ensemble model. This model is made up of three distinct models (CNN, LSTM, and LSTM-BLSTM). First, we present the individual classifiers’ classification results. The performance of the ensemble-based model is then evaluated. We adopted the majority voting ensemble (MVE) because it is the most widely used approach in the research area because it avoids the limitations of other techniques mentioned previously, and it also performs well in many approaches. The results of the proposed models are compared to each other. Furthermore, a comparison to the results of previous work [13] is made.

Tables 6 and 7 compare our proposed models to each other in order to assess their performance from various perspectives, whereas Tables 8 and 9 compare our work to the previous study [13] using the same metrics found in their paper. Tables 6 and 7 show an evaluation of all proposed models for 6 months and 12 months’ data in terms of Precision, Sensitivity, F1-score, Specificity, and Accuracy. These values are shown in the detailed results for CKD and Non-CKD separately, as well as the macro and weighted averages. Tables 8 and 9 show a comparison of the models proposed in this study and previous work [13] on the same datasets in terms of Precision, Sensitivity, Specificity, F1-score, and Accuracy. The values in bold font represent the best accuracy achieved in the compared models. These results show that the ensemble model outperforms the individual models and previous work results in many aspects: sensitivity, precision, specificity, F1-score and accuracy. The proposed model has proven its worthiness in all these respects.

**Table 6:** Results for 6_months prediction data produced by our proposed models

Model	Precision				Sensitivity				F1-score				Accuracy
	CKD	Non-CKD	Macro avg	Weighted avg	CKD	Non-CKD	Macro avg	Weighted avg	CKD	Non-CKD	Macro avg	Weighted avg	
Proposed models													
CNN_model_1	0.95	0.89	0.92	0.94	0.98	0.79	0.88	0.94	0.96	0.84	0.90	0.94	0.94
LSTM_model_1	0.95	0.87	0.91	0.94	0.97	0.80	0.89	0.94	0.96	0.84	0.90	0.94	0.94
Individual classifiers in Ensemble model													
CNN_model_2	0.97	0.95	0.96	0.96	0.99	0.86	0.92	0.96	0.98	0.90	0.94	0.96	0.9626
LSTM_model_2	0.99	0.97	0.98	0.98	0.99	0.95	0.97	0.98	0.99	0.96	0.97	0.98	0.9825
LSTM-BLSTM	0.99	0.98	0.98	0.98	1.00	0.94	0.97	0.98	0.99	0.96	0.98	0.98	0.9849
Ensemble model													
Ensemble model	**0.99**	**1.00**	**1.00**	**0.99**	**1.00**	**0.97**	**0.98**	**0.99**	**1.00**	**0.98**	**0.99**	**0.99**	**0.9931**

**Table 7:** Results for 12_months prediction data produced by our proposed models

Model	Precision				Sensitivity				F1-score				Accuracy
	CKD	Non-CKD	Macro avg	Weighted avg	CKD	Non-CKD	Macro avg	Weighted avg	CKD	Non-CKD	Macro avg	Weighted avg	
Proposed models													
CNN_model_1	0.95	0.87	0.91	0.93	0.97	0.79	0.88	0.93	0.96	0.83	0.89	0.93	0.93
LSTM_model_1	0.95	0.88	0.92	0.94	0.97	0.80	0.89	0.94	0.96	0.84	0.90	0.94	0.94
Individual classifiers in Ensemble model													
CNN_model_2	0.96	0.95	0.95	0.95	0.99	0.82	0.91	0.95	0.97	0.88	0.93	0.95	0.954
LSTM_model_2	0.98	0.95	0.97	0.98	0.99	0.93	0.96	0.98	0.99	0.94	0.96	0.98	0.977
LSTM-BLSTM	0.99	0.97	0.98	0.99	0.99	0.96	0.98	0.99	0.99	0.97	0.98	0.99	0.987
Ensemble model													
Ensemble model	**0.99**	**1.00**	**0.99**	**0.99**	**1.00**	**0.97**	**0.98**	**0.99**	**1.00**	**0.98**	**0.99**	**0.99**	**0.992**

**Table 8:** Comparison of performance metrics for 6-month data obtained from the proposed models and the literature

Model	Precision	Sensitivity	Specificity	F1-score	Accuracy
Proposed models					
CNN_model_1	0.94	0.94	0.88	0.94	0.94
LSTM_model_1	0.94	0.94	0.87	0.94	0.94
Individual classifiers in Ensemble model					
CNN_model_2	0.96	0.96	0.94	0.96	0.9626
LSTM_model_2	0.98	0.98	0.96	0.98	0.9825
LSTM-BLSTM	0.98	0.98	0.98	0.98	0.9849
Ensemble model					
CNN-LSTM-LSTM-BLSTM	**0.99**	**0.99**	**0.99**	**0.99**	**0.9931**
Results for the previous work [13]					
LightGBM [13]	0.426	0.685	0.767	0.525	0.751
Logistic [13]	0.405	0.664	0.754	0.503	0.736
Random forest [13]	0.390	0.652	0.743	0.488	0.725
Decision tree [13]	0.395	0.622	0.76	0.483	0.732

**Table 9:** Comparison of performance metrics for 12-month data obtained from the proposed models and the literature

Model	Precision	Sensitivity	Specificity	F1-score	Accuracy
Proposed models					
CNN_model_1	0.93	0.93	0.86	0.93	0.93
LSTM_model_1	0.94	0.94	0.88	0.94	0.94
Individual classifiers in Ensemble model					
CNN_model_2	0.95	0.95	0.94	0.95	0.954
LSTM_model_2	0.98	0.98	0.95	0.98	0.977
LSTM-BLSTM	0.99	0.99	0.97	0.99	0.987
Ensemble model					
CNN-LSTM-LSTM-BLSTM	**0.99**	**0.99**	**0.99**	**0.99**	**0.992**
Results for the previous work [13]					
LightGBM [13]	0.437	0.654	0.786	0.524	0.759
Logistic [13]	0.39	0.66	0.738	0.491	0.722
Random forest [13]	0.406	0.608	0.774	0.487	0.74
Decision tree [13]	0.399	0.604	0.77	0.48	0.735

**Fig. 6:**
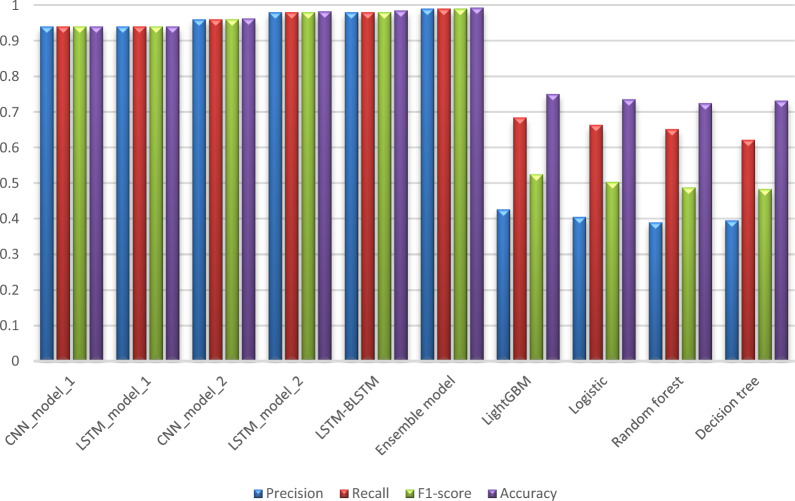
Comparison of performance metrics for 6-month data

**Fig. 7:**
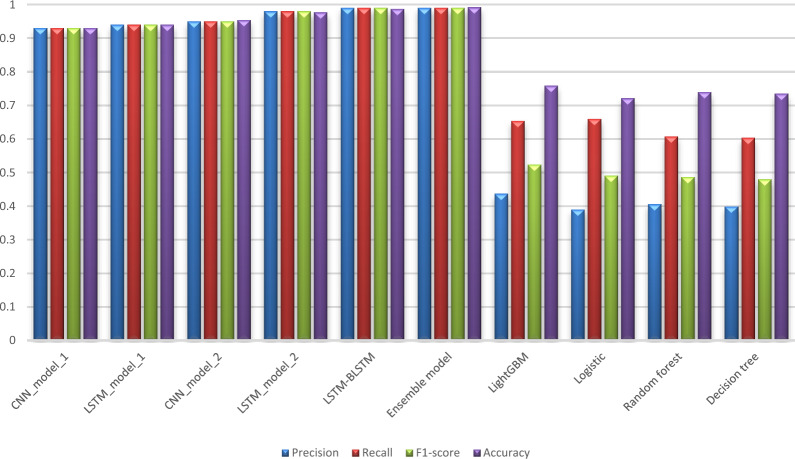
Comparison of performance metrics for 12-month data

Figures 6 and 7 display a graphical representation of the performance of all proposed models as well as the models in the comparative paper [13] on the same datasets for both 6 months and 12 months’ data. The figures show the model’s performance improvement over previous models.

**Fig. 8:**
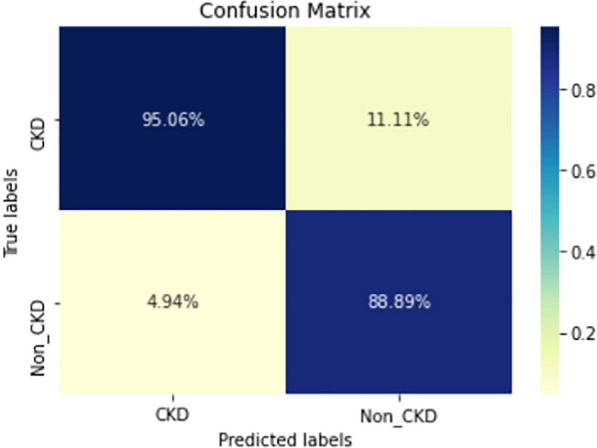
Confusion matrix of CNN model_1 (6_months data)

**Fig. 9:**
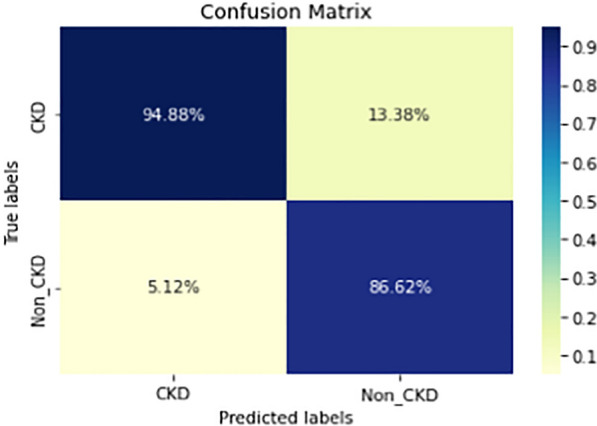
Confusion matrix of CNN model_1 (12_months data)

**Fig. 10:**
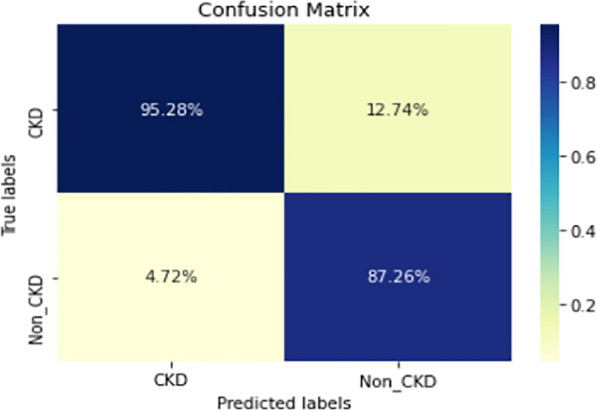
Confusion matrix of LSTM model_1 (6_months data)

**Fig. 11:**
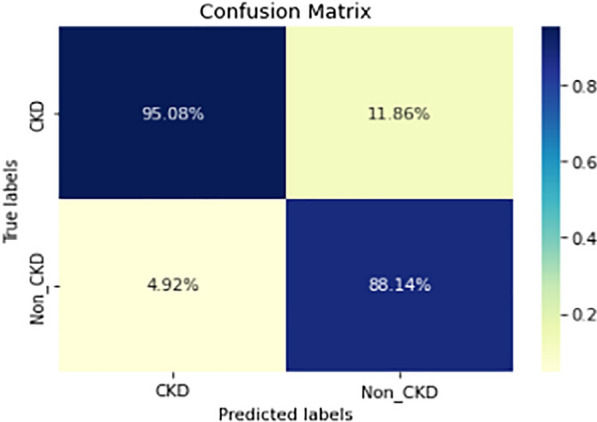
Confusion matrix of LSTM model_1 (12_months data)

**Fig. 12:**
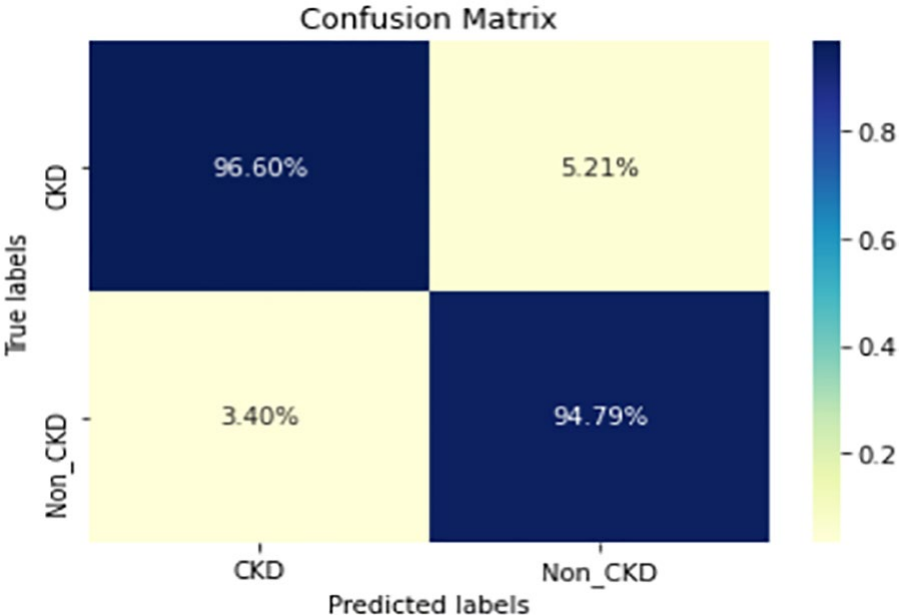
Confusion matrix of CNN model_2 (6_months data)

**Fig. 13:**
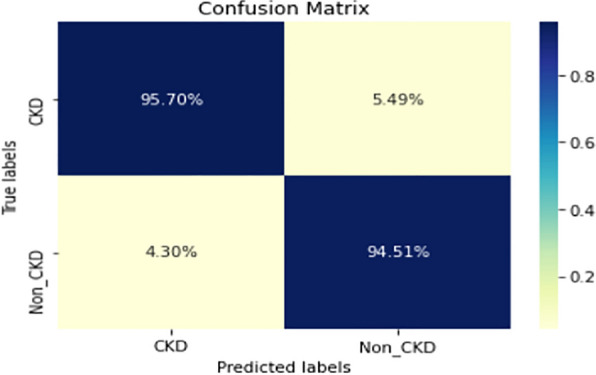
Confusion matrix of CNN model_2 (12_months data)

**Fig. 14:**
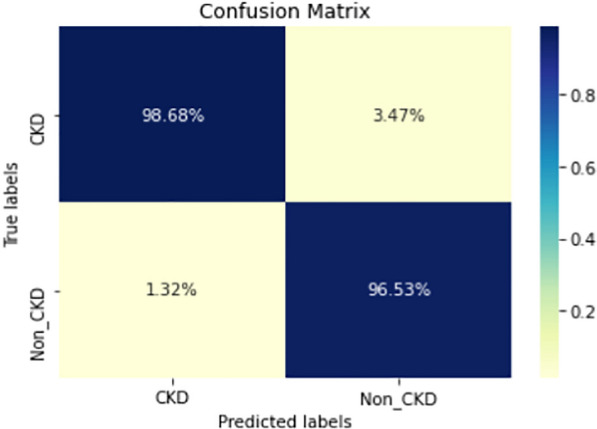
Confusion matrix of LSTM model_2 (6_months data)

**Fig. 15:**
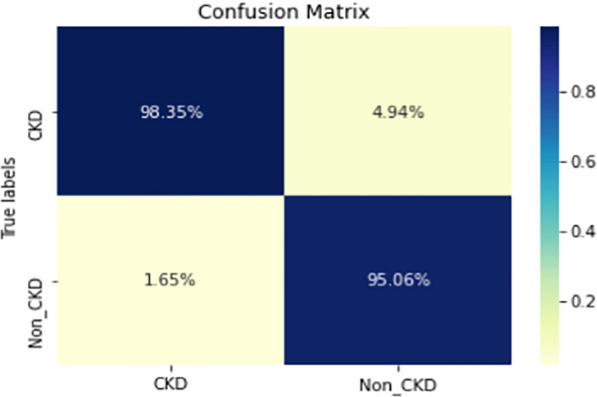
Confusion matrix of LSTM model_2 (12_months data)

**Fig. 16:**
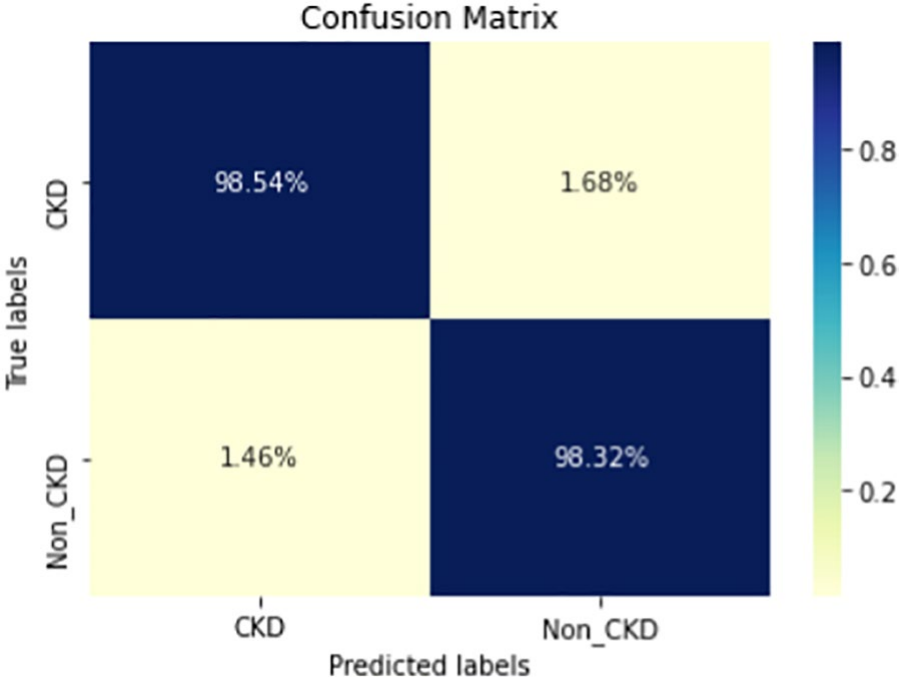
Confusion matrix of LSTM-BLSTM model (6_months data)

**Fig. 17:**
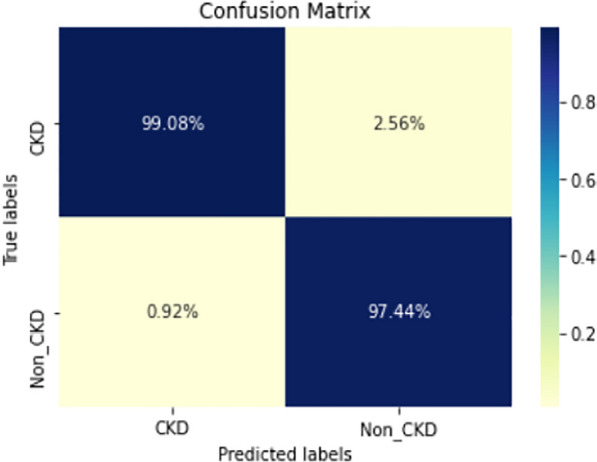
Confusion matrix of LSTM-BLSTM model (12_months data)

**Fig. 18:**
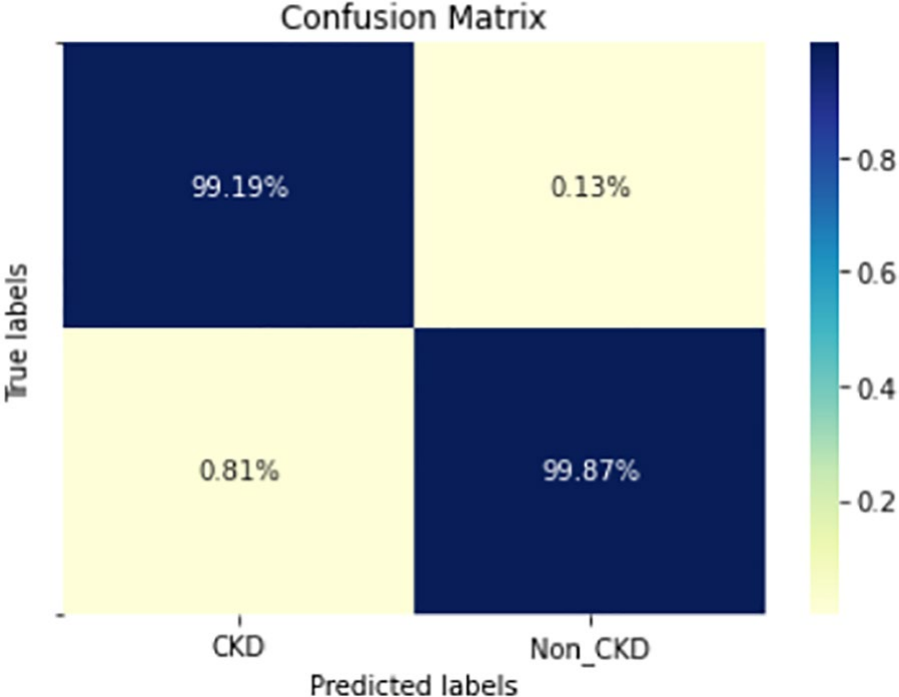
Confusion matrix of Ensemble model (6_months data)

**Fig. 19:**
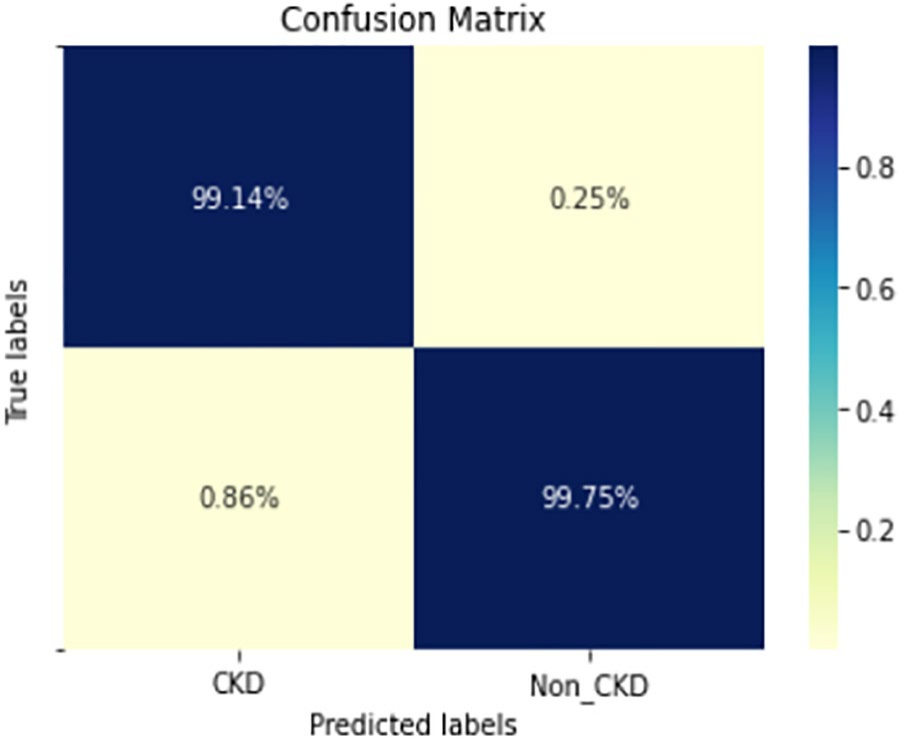
Confusion matrix of Ensemble model (12_months data)

Figures  8, 9, 10, 11, 12, 13, 14, 15, 16, 17, 18 and 19 show the confusion matrices for the predictive models, which represent the confusion matrix results for 6 months and 12 months’ data, respectively. The figures demonstrate the model’s classification robustness in the two statuses, CKD and Non-CKD. Finally, Figs. 18 and 19 show the ensemble model’s confusion matrix results. This model’s strength and outstanding performance in many types of research also demonstrate its worthiness in our case. Unfortunately, this model requires more memory space and takes longer than the individual models, indicating that its computational complexity is higher than the others. However, its accuracy outperforms that of the individual models.

### Results and discussion

The main objective of the proposed research is the classification of CKD and Non-CKD classes with higher classification performance. Recently, the number of publicly available CKD datasets has become limited and contain only a small number of samples. Hence, the use of existing datasets with a large number of samples strongly supports the performance of the models. The main advantage of this method is that it does not need laboratory data as related studies in this field. However, it necessitates demographic information such as age and gender, in addition to prescriptions and diagnoses from patients. This information is widely available and easy to obtain from the appropriate authorities.

The experimental results show that the accuracy of the (LSTM_BLSTM) model outperforms all other individual deep learning models, as shown in Tables 6 and 7 for each individual model, with an accuracy score of 98.49%, 98.7% for 6 months and 12 months’ data, respectively. Furthermore, the confusion metrics show that the BLSTM-LSTM model is capable of classifying CKD and Non-CKD cases with 98.54%, 98.32% for 6 months’ data and 99.08%, 97.44% for 12 months’ data, respectively. The findings of the comparison show that the LSTM model outperforms the CNN model. For 6 months and 12 months’ data, the accuracy of the second CNN model increased by 2.26% and 2.4%, respectively, over the accuracy of the first CNN model. As we utilized conv_2D in the first CNN model and conv_1D in another, modifying the structure of the model enhances the outcomes of the base models and produced better results. Furthermore, we employ the LSTM model separately and integrate it with the BLSTM model to create a hybrid model, which also enhanced the results. Finally, combining the best models to create an ensemble model enhances the overall performance. It is also clear that using the ensemble approach leads to significant improvements in the model’s prediction capability, as the results show that the performance using the proposed ensemble model that combines (CNN, LSTM, and LSTM_BLSTM) models outperformed all other models significantly, with an accuracy score of 99.31% for 6 months, and 99.2% for 12 months, respectively. This is accomplished by strengthening the classification step with the ensemble algorithm’s majority voting capability, which increases prediction accuracy. As a result, our findings suggest that the proposed ensemble model has improved complex feature representations and will perform well in the classification task.

According to previous research [13], the LightGBM achieved the highest accuracy among the other machine learning techniques on the various aggregated datasets, with 0.751 and 0.759 for 6 months and 12 months’ data, respectively. Deep learning techniques, as shown in Tables 8 and 9, significantly improved accuracy in our study. While using the ensemble method has greatly improved the results. Figures 6 and 7 demonstrate a summary of the comparison with literature-based approaches developed for CKD prediction using the same dataset. This method is appropriate for a population study but not for assisting clinicians with individual patients. To achieve the best results in patient diagnosis, decisions should be based on laboratory tests [13].

The following are the key features of the proposed research in CKD prediction:

- Our proposed models, which are based on deep learning models, can effectively predict CKD 6 or 12 months before its occurrence, which is considered a scientific breakthrough and contributes to saving people’s lives.
- Deep learning techniques outperform traditional machine learning approaches in terms of performance because of their ability to extract features from input data without the intervention of a human. As a result, a better classification model is produced.
- If the model parameters are well designed and optimized, changing the structure of the CNN and LSTM models leads to better results.
- A hybrid model (LSTM_BLSTM) outperforms all other individual deep learning models with an accuracy score of 98.49%, for 6 months, and 98.7% for 12 months, which is higher than the existing recent models.
- When compared to traditional machine learning approaches and individual deep learning models, using an ensemble model can significantly improve accuracy.

## Conclusion

Recently, machine learning research has shown that combining the output of several individual classifiers can reduce generalization errors and yield better performance in many applications than individual deep learning classifiers. As a result, the Ensemble learning algorithm has become dependable and dominant in a variety of fields. The Ensemble model’s main idea is to train multiple models and then combine their predictions using one of the combination techniques. In the case of kidney disease, scientists have attempted to detect it early or predict its occurrence. The practical implications of this research are that most previous studies have focused on disease detection, while only a few have focused on disease prediction before it occurs. Furthermore, the previous model’s low accuracy. Given the value of human life, developing a more accurate model leads to faster intervention to save patients’ lives.

This study focuses on Chronic Kidney Disease prediction within 6 or 12 months earlier based on medication, demographic, and comorbidity data from patients using the Deep Ensemble algorithm, which is considered a breakthrough in the scientific field. This research was conducted using two different public benchmark datasets obtained from Taiwan’s National Health Insurance Research Database. We propose three predictive models in this study: CNN model, LSTM model and the Deep Ensemble model fuses three base deep learning classifiers (CNN, LSTM, and LSTM-BLSTM) using the majority voting technique to improve classification performance.

We separately assessed the performance of the three proposed predictive models. Furthermore, we compared the proposed Deep Ensemble model’s performance to that of each base deep learning classifier. Finally, we compared the proposed models’ results to the results of the comparative paper, which used the same datasets and traditional machine learning techniques. According to the findings of this study, the proposed models performed significantly better. It can also be deduced that the proposed deep ensemble model that combines (CNN, LSTM, and LSTM_BLSTM) significantly outperforms the approaches mentioned in the literature and the deep learning models proposed in this study, with accuracy scores of 99.31%, 99.2% for 6 months and 12 months, respectively. Furthermore, the superiority of this model is distinctive.

The limitation is that this approach is appropriate for a population study but not for assisting clinicians with individual patients. To achieve the best results in patient diagnosis, decisions should be based on laboratory tests [13]. This approach, on the other hand, sheds light on previously unknown features for CKD prediction. Furthermore, due to a lack of these features in the dataset, the risk factors for this disease, such as a family history of kidney failure, hypertension, and diabetes, were not determined in this study. Finally, our deep learning models require more memory storage and a longer learning time than traditional machine learning techniques. As a result, the ensemble model needs more memory and extended time than deep learning models, as each run separately and then gathers into the proposed model.

In the future, we plan to test the robustness of our developed models against various datasets based on patient laboratory data collected from local hospitals, medical analysis laboratories, and polyclinics. Furthermore, we intend to broaden our research to include more classes of CKD detection, not just prediction, such as first and second stage CKD. Using another dataset, we can also determine the risk factors for CKD, such as a family history of kidney failure, hypertension, and diabetes.

## Data Availability

The datasets analysed during the current study are available in a public repository [23].
